# Dispositional mindfulness moderates the relation between neuroticism and depressive symptoms

**DOI:** 10.1016/j.paid.2011.07.032

**Published:** 2011-12

**Authors:** Thorsten Barnhofer, Danielle S. Duggan, James W. Griffith

**Affiliations:** a Department of Psychiatry, University of Oxford, United Kingdom; b Northwestern University, Feinberg School of Medicine, Department of Medical Social Sciences, United States

**Keywords:** Mindfulness, Neuroticism, Depression, Moderator, Cognitive reactivity

## Abstract

Negative emotional reactivity as measured by neuroticism has been shown to be an important risk factor for the development of depressive symptoms. This study investigated whether the ability to be mindful can protect against the negative effects of this temperamental vulnerability. An English community sample of *N* = 144 individuals who had completed a neuroticism questionnaire six years previously were assessed for current depressive symptoms and dispositional levels of mindfulness at points of assessment approximately one year apart. Dispositional mindfulness moderated the relation between neuroticism and current depressive symptoms: Neuroticism was significantly related to depression in those with low to medium levels of dispositional mindfulness but not in those with relatively high levels of mindfulness. Further analyzes focusing on particular mindfulness skills indicated that this effect was carried mostly by the ability to describe inner experience. The results suggest that dispositional mindfulness and particularly the ability to describe inner experience are helpful in dealing with negative emotional reactivity in a way that reduces the likelihood of depressive symptoms to develop.

## Highlights

► We examined the effects of mindfulness on the relation between neuroticism and depression. ► Neuroticism predicts current depressive symptoms even when assessed six years apart. ► Dispositional mindfulness moderates this relationship. ► High levels of dispositional mindfulness protect against negative effects of neuroticism.

## 1 Introduction

Neuroticism, a stable temperament that arises early in life, is one of the best-established vulnerability factors for depression (Kotov, Gamez, Schmidt, & Watson, 2010). High levels of neuroticism are associated with an increased overall risk of depression (e.g. Kendler, Gatz, Gardner, & Pedersen, 2006), an increased likelihood that occurrence of stressful life events will lead into depressive disorder (Ormel, Oldehinkel, & Brilman, 2001), and a more protracted course in those who have become depressed (Hirschfeld, Klerman, & Andreasen, 1986). Neuroticism is a complex construct that includes several different traits and facets (see Eysenck & Eysenck, 1985), including thinking styles such as being “irrational”, and denotes an increased general tendency towards negative emotional reactivity and arousal.

There is evidence that the relation between neuroticism and depressive symptoms is mediated by ruminative tendencies and increased cognitive reactivity, which is defined as the tendency for negative thinking to become triggered through only subtle changes in mood (Barnhofer & Chittka, 2010; Roelofs, Huibers, Peeters, Arntz, & van Os, 2008). Ruminative tendencies and cognitive reactivity both play an important role in the recurrence and maintenance of depressive symptoms and are therefore important targets for preventative interventions (Nolen-Hoeksema, Wisco, & Lyubomirsky, 2008; Scher, Ingram, & Segal, 2005). Recently interest has increased in the use of training in mindfulness meditation as a way of addressing these factors. Mindfulness has been described as the ability to maintain awareness moment by moment in an open and acceptant way (Kabat-Zinn, 2003). Importantly for clinical care, training in mindfulness can help individuals become better able to identify and disengage from maladaptive patterns of responding and thus prevent downward spirals of negative mood and thinking (e.g. Segal, Williams, & Teasdale, 2002). Other research on mindfulness-based interventions lends further support: In those who are at risk for depression, intensive training in mindfulness reduces ruminative tendencies (Ramel, Goldin, Carmona, & McQuaid, 2004) and the negative effects of cognitive reactivity (Kuyken et al., 2010). Rumination and cognitive reactivity are processes that are high in people who are high in neuroticism, so if mindfulness can reduce these processes, it seems plausible that mindfulness is a skill that can help to prevent neuroticism from translating into depressive symptoms. Thus, delineating such effects would be helpful in understanding how the negative emotional outcomes of neuroticism can be prevented. This would be important for the prevention of depression, as well as the broad range of emotional disorders given that neuroticism accounts for a significant amount of common variance across the mood and anxiety disorders (Griffith et al., 2010). Mindfulness-based interventions are now increasingly being adapted for the whole spectrum of these disorders (Hofmann, Sawyer, Witt, & Oh, 2010) and demonstrating the effects on global vulnerability factors would be an important step in justifying such broadening of application.

Clinical applications focus on mindfulness as a trainable skill, but it can also be conceptualized as a dispositional variable that can be assessed using self-report questionnaires (Baer, Smith, Hopkins, Krietemeyer, & Toney, 2006). Although such self-reports are sensitive to changes following intensive training in mindfulness, there is also evidence that without such training levels of mindfulness remain relatively stable over time (Baer, Smith, & Allen, 2004; Brown & Ryan, 2003). That is, individuals seem to differ in their natural tendency to be aware of their moment to moment experience in an open and non-judgmental way. Validation studies have related self-reports of mindfulness to a range of behavioral and cognitive variables reflecting hypothesized consequences of mindfulness. For example, event sampling studies have shown that self-reported mindfulness predicts higher levels of autonomy and lower levels of unpleasant affect in daily functioning (Brown & Ryan, 2003). A recent brain study has demonstrated that self-reported levels of dispositional mindfulness are related to resting activity in brain areas involved in self-referential processing as well as amygdala reactivity when viewing emotional faces (Way, Creswell, Eisenberger, & Lieberman, 2010).

Consistent with the assumption that mindfulness may protect against the negative effects of emotional vulnerabilities, dispositional mindfulness is negatively related to neuroticism (Giluk, 2009). Furthermore, there is some evidence that it may offset its negative effects. Feltman, Robinson, and Ode (2009) assessed dispositional mindfulness, neuroticism and depressive symptoms cross-sectionally in a sample of students and found that dispositional mindfulness moderated the relation between neuroticism and depressive symptoms: Neuroticism was significantly related to depressive symptoms in those with low levels of dispositional mindfulness, but there was no significant relation between neuroticism and depressive symptoms in those with high levels of dispositional mindfulness. The current study was aimed at replicating and extending these findings.

For this study an opportunity had arisen to test the protective effects of dispositional mindfulness in a general population sample that provided information on neuroticism six years before our assessment of depressive symptoms and dispositional mindfulness – also at separate occasions. Investigating relations over relatively remote points in time is consistent with the idea that neuroticism functions as a relatively stable temperamental risk factor and also allowed us to provide stronger control against the effects of general response bias. Previous research on this sample had shown a significant correlation between neuroticism scores assessed six years earlier and current symptoms of depression (Barnhofer & Chittka, 2010). Extending this research in this sample, we hypothesized that when taking into account dispositional mindfulness this relationship would remain significant in those low in dispositional mindfulness but not in those high in dispositional mindfulness.

We had used the Five Factor Mindfulness Questionnaire (FFMQ; Baer et al., 2006) to assess dispositional mindfulness, which describes mindfulness as a global factor that encompasses several distinguishable skills. Subscales of this questionnaire measure an individual’s ability to observe internal and external experience, to describe internal experience, to act with awareness, to be non-judgmental, and to be non-reactive to inner experience. Analyzes based on these subscales allowed us to explore which mindfulness skills might be most relevant in offsetting the effects of neuroticism.

## 2 Methods

### 2.1 Participants

Participants for this study were recruited from a large randomly-ascertained family cohort in southwest England (*N* = 88,000; Martin et al., 2000) who had given their written permission to be contacted for participation in further research. Participants had provided information on neuroticism 6 years before they were approached for the current study. Data on depression and mindfulness were collected in separate assessments. In an initial step, 707 potential participants received letters about the study. A subset of these (223, 32%) indicated their willingness to take part and were sent a booklet including a questionnaire assessing current symptoms of depression along with an informed consent form and a stamped return envelope. A subset of these participants (182, 81%) returned the questionnaire booklet with their signed consent form. They were then contacted approximately one year later to ask them to complete further questionnaires, including the measure of mindfulness. The final sample is the 144 participants (79% of the previous respondents) who returned this second set of questionnaires together with the consent form. The average age of this final sample was *M* = 43.0 (*SD* = 6.8, age range: 27–59) years. Eighty-seven (60%) of them were women, 57 (40%) of them were men. Six (4%) of the participants reported regularly using a meditation or related technique. However, none of them engaged in mindfulness meditation (3 practised Christian prayer meditation, 1 yogic breathing, 1 creative visualization, and 1 transcendental meditation). The studies had received ethical approval from the Oxfordshire Psychiatric Research Ethics Committee and the University of Oxford Ethics Committee.

### 2.2 Measures

The first questionnaire booklet sent to participants included the Beck Depression Inventory-II (BDI-II). The Five Factor Mindfulness Scale was included in the second questionnaire booklet that was sent one year after the first. Six years before we first re-contacted the sample, neuroticism had been assessed as part of a larger community-based study using commercial mailing in which participants were sent the Eysenck Personality Questionnaire to complete at home and return via mail.

#### 2.2.1 Eysenck Personality Questionnaire (EPQ)

The EPQ (Eysenck & Eysenck, 1975) is a self-report questionnaire consisting of 90 items with a binary response format. The neuroticism scale of the EPQ consists of 23 items. Internal consistencies in the current sample for all questionnaires are listed in Table 1. Eysenck and Eysenck (1975) report a test-retest reliability over one month of *r* = .89.

#### 2.2.2 Beck Depression Inventory-II (BDI-II)

The BDI-II (Beck, Steer, & Brown, 1996) contains 21 statements that assess the severity of depressive symptoms such as low mood, anhedonia, changes in sleep, appetite, concentration, etc. over the preceding two weeks. Beck et al. (1996) report good internal consistency in both patient and student samples and one-week re-test-reliability of *r* = .93 suggesting that the test is robust against daily variations in mood in depressed samples.

#### 2.2.3 Five Factor Mindfulness Questionnaire (FFMQ)

The FFMQ (Baer et al., 2006) was developed based on factor analyzes of previously published mindfulness questionnaires. It assesses five facets of a general tendency to be mindful in daily life: observing (“I notice the smells and aromas of things”), describing (“I am good at finding words to describe my feelings”), acting with awareness (“I find myself doing things without paying attention” – reverse scored), non-judging of inner experience (“I think some of my emotions are bad or inappropriate and I should not feel them” – reverse scored), and non-reactivity to inner experience (“I perceive my feelings and emotions without having to react to them”). In line with the assumption that mindfulness has beneficial effects on emotional health, validation studies have reported negative correlations between the FFMQ (total and subscale scores) and self-report measures of emotional symptoms and distress as well as positive correlations with self-report measures of psychological well-being (Baer et al., 2008). Internal consistency of the subscales of the FFMQ in our sample was generally acceptable (see Table 1).

## 3 Results

Zero-order correlations showed that neuroticism scores assessed 6 years previously were correlated with the severity of current symptoms of depression as assessed by BDI-II, *r* = .56, *p* < .001. The FFMQ total mindfulness score was inversely correlated with both neuroticism, *r* = −.60, *p* < .001, and severity of current symptoms of depression, *r* = −.58, *p* < .001. Correlations of the subscales of the FFMQ showed the same pattern of findings – significant inverse correlations with both neuroticism and current symptoms of depression – for all of the subscales apart from the “Observing” scale, which did not show a significant relation with either neuroticism or severity of current symptoms of depression. Correlation coefficients, means and standard deviations of raw scores and percent of maximum possible scores (POMP; Cohen, Cohen, Aiken, & West, 1999) on all scales are listed in Table 1.

In order to investigate the effects of neuroticism and mindfulness on current symptoms of depression we conducted a linear regression. In the first step EPQ neuroticism was entered as predictor of BDI-II scores yielding a significant effect, *t* = 8.21, *p* < .001, *β* = .56, *R*^2^ = .32, ƒ^2^ = .47. The FFMQ sumscore was then entered as an additional predictor in the second step, which showed a significant effect of mindfulness over and above the effects of neuroticism, *t* = −4.51, *p* < .001, *β* = −.36, *R*^2^ change = .09, ƒ^2^ = .10, with higher scores in mindfulness being related to lower current depression. Finally, to test the interaction between neuroticism and mindfulness, the product of centered EPQ neuroticism and centered FFMQ sumscores was entered as an additional predictor in the third step. In line with our hypothesis, the interaction between neuroticism and mindfulness emerged as a significant predictor, *t* = −2.49, *p* = .01, *β* = −1.00, *R*^2^ change = .03, ƒ^2^ = .03.

**Fig. 1:**
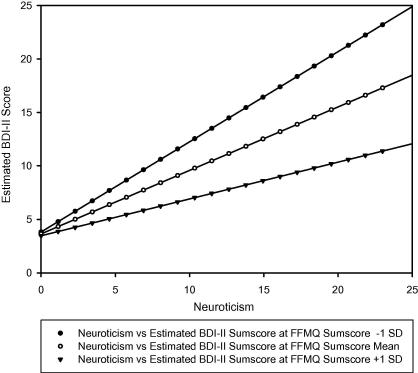
Regression lines of the relation between EPQ Neuroticism and BDI-II scores at high (+1 *SD* = 100.24), medium (*M* = 120.92) and low levels (−1 *SD* = 141.60) of dispositional mindfulness (FFMQ sumscore).

Fig. 1 illustrates the interaction by depicting the regression lines of the relation between neuroticism and current depression at high, medium and low (+1 SD, mean, −1 SD) scores of the FFMQ sumscore scale. Decreases in the slope of the regression line with increasing mindfulness scores show that the relation between neuroticism and current symptoms of depression becomes weaker with higher levels of dispositional mindfulness.

In order to further characterize the nature of this interaction we used the Johnson–Neymann (J–N) technique (following suggestions and using the SPSS script provided by Hayes & Matthes, 2009). The J–N technique allows to directly identify points in the range of the moderator variable where the effect of the predictor on the outcome transitions from being statistically significant to nonsignificant by finding the value of the moderator variable for which the ratio of the conditional effect to its standard error is equal to the critical *t* score. The conditional effect of neuroticism on current depression transitioned in significance at a FFMQ sumscore of 145.51, *b* = .30, *SE* = .15, *t* = 1.97, *p* = .05, 95% CIs [.00, .60], at the 90th percentile of the distribution in our sample, with the relation between EPQ neuroticism and BDI-II scores significant at FFMQ sumscores below this threshold and nonsignificant at FFMQ sumscores above this threshold.

In order to further investigate which components of mindfulness skills were most relevant in moderating the effects of neuroticism on current depression, we repeated the above analyzes separately with all five subscales of the FFMQ. After adjusting α-levels for familywise error rate to *α* = .01, none of the interactions were significant. The only interaction that approached significance was for the Describing subscale, interaction neuroticism by FFMQ Describing: t = −2.88, p = .02, β = −.66, R^2^ change = .02, f^2^ = .03. Probing this effect using the J–N technique showed that significance at the .05 level transitioned at a score of 37.01, *b* = .40, *SE* = .20, *t* = 1.97, *p* = .050, 95% CIs [.00, .80], the 93rd percentile of the distribution in our sample with the pattern of the effect following that of the effect for the FFMQ sumscore, i.e. the conditional effect of neuroticism on current depression being significant below and nonsignificant above the threshold. As most of the subscales of the FFMQ are moderately intercorrelated (intercorrelations in our sample ranged from *r* = .08 to .54), we also ran a regression analysis including all FFMQ subscales and their interactions with neuroticism simultaneously in order to estimate unique contributions of the Describing subscale by neuroticism interaction. Controlling for the contribution of other subscales and their interactions with neuroticism, the interaction of the Describe subscale with neuroticism approached significance, *t* = −1.93, *p* = .056, *β* = −.68, all other interactions *p* > .60.

Current meditation practice was not significantly related to trait mindfulness, *r* = .12, *p* = .13, nor did results of the regression analyzes change substantially when current practice and its interaction were entered as covariates.

## 4 Discussion

The current study showed that, even when assessed several years earlier, neuroticism can significantly and strongly predict depressive symptoms later in time. Consistent with our hypotheses, dispositional mindfulness moderated this relationship. The higher an individual’s level of dispositional mindfulness, the weaker the relation between neuroticism and depressive symptoms. That is, in those with high levels of dispositional mindfulness, neuroticism seemed to be less likely to translate into the occurrence of negative emotional outcomes in the shape of depressive symptoms. These findings are in line both with results from previous studies in students (Feltman et al., 2009) and clinical findings that show that increases in mindfulness following meditation training can reduce engagement in maladaptive cognitive processes related to neuroticism (Kuyken et al., 2010; Ramel et al., 2004). These findings also suggest that dispositional mindfulness may act as a protective factor against the effects of negative emotional reactivity indexed by neuroticism. However, it is important to highlight from the beginning of the discussion that this effect was small. Nevertheless, the fact that we were able to replicate results of an earlier study in a design relating assessments from different points in time increases confidence in the finding of the moderating effects of dispositional mindfulness. The current results are less likely to be influenced by general response biases, which can easily play a larger role when measures of temperament and measures of symptoms are assessed at the same point in time.

The current study has a number of limitations. Firstly, the findings are based solely on self-report and therefore potentially suffer from reporting biases. It is also important in this regard to highlight that there is currently debate about whether relevant aspects of mindfulness can be accessed via self-report. A crucial question in this context is whether it is possible to systematically relate self-reports of mindfulness to more objective behavioral or biological indicators of mindfulness and its consequences (Davidson, 2010). As described in the introduction, currently accumulating findings are encouraging in this regard and suggest that self-reports of the ability to be mindful may at least provide a viable first step into the interrogation of relations between mindfulness and emotional outcomes. Secondly, because levels of mindfulness and depressive symptoms were assessed at different points in time, interpretation of our findings rests on the assumption that FFMQ scores remained stable and that they were unaffected by prior symptoms of depression. There is currently no information available on the test-retest reliability of the FFMQ. However, there is evidence that other mindfulness questionnaires, which provided items for the FFMQ, show good test-retest reliability (Kentucky Inventory of Mindfulness Skills; Baer et al., 2004, Mindful Attention Awareness Scale; Brown & Ryan, 2003) and it seems plausible to assume that the FFMQ is likely to perform similarly to its constituent measures. Thirdly, it is not possible to rule out effects of unassessed third variables that might have impacted on the observed relations and, indeed, it is quite plausible that the observed relations are carried by more proximal variables that are known to mediate the relation between neuroticism and depressive symptoms such as rumination or cognitive reactivity (Barnhofer & Chittka, 2010). In the absence of baseline measures of depression it is not possible to estimate in how far the observed relations between neuroticism and later depression were carried by persisting levels of depression. Fourthly, because symptoms of depression and trait mindfulness were assessed at points of time one year apart it is possible that levels of mindfulness might have changed as a response to prior depression. However, we were able to rule out influences of meditation practice as none of the participants had engaged in mindfulness meditation or received mindfulness-based therapy for relapse prevention and engagement in other meditation practices did not affect the observed relations.

Despite these limitations the current findings provide a number of insights. Moderating effects of mindfulness on the translation of temperamental risk into negative emotional outcomes are interesting from a clinical point of view given the very different nature of the constructs involved. Whereas neuroticism mainly reflects negative emotional sensitivity and reactivity, dispositional mindfulness indexes attentional skills and attitudes guiding the way in which individuals relate to inner experience. The relations found here are therefore unlikely to be due simply to conceptual overlap between constructs and speak directly to how training of attentional processes may influence the effects of temperamental vulnerabilities.

The results of analyzes on the effects of different facets of mindfulness skills only approached significance and can only be interpreted with great caution perhaps serving as pointers for future research to be conducted. They suggest Describing to be the most relevant of mindfulness skills in the moderation of neuroticism outcomes. Describing indexes the ability to consciously note and label current experience. Although on the surface, it might simply occur as a passive way of registering experience, there is evidence that this strategy can significantly facilitate the regulation of negative emotions. Neuroscientific studies have shown that the labeling of affective states activates a top-down regulatory mechanism in which limbic activity is inhibited through activation of prefrontal areas of the brain and that this effect is increased in individuals with high levels of dispositional mindfulness (Creswell, Way, Eisenberger, & Lieberman, 2007). The current results point towards the possibility that the verbal labeling of experience and the conscious noting and recognizing of mental and bodily events that comes with it may be at the heart of the decentering mechanisms through which mindfulness is assumed to exert its effects (Teasdale, 1999).

At what levels of dispositional mindfulness do such protective effects become evident? Probing the interaction between neuroticism and mindfulness, we found that the significance of the relation between neuroticism and current depressive symptoms turned at an FFMQ sumscore of 145.5, which within our sample was located at the 90th percentile of the distribution. The negative effects of neuroticism thus seem to become offset only at relatively high levels of dispositional mindfulness, a finding that may also speak to why the effects observed here were relatively small. Interestingly, the level at which the moderating effect of mindfulness occurred is almost identical to the mean mindfulness score previous validation research has reported for longterm meditators (Baer et al., 2008) suggesting that in order to reach levels of mindfulness that have protective effects most individuals would indeed have to engage in sustained training of meditation.

## 5 Conclusions

The current research is relevant to treating the emotional disorders. It is well known that emotional disorders share common symptoms and variance (Krueger, 1999), and this common variance strongly overlaps with neuroticism (Griffith et al., 2010). It has been suggested that the mental skills reflected by the construct of mindfulness may help to counter global vulnerabilities for emotional disorders (Williams, 2008). Protocol-driven interventions that focus on core emotional symptoms have emerged and are currently being studied and used in clinical settings (e.g. Allen, McHugh, & Barlow, 2008), and the inclusion of mindfulness training in these protocols has the potential to further enhance treatment outcome. The current findings support the therapeutic potential of mindfulness. They suggest that high levels of dispositional mindfulness can protect against the negative effects of neuroticism. The ability to describe and label inner experience is likely to be a particularly important skill in this context.

Further research will have to demonstrate similar effects for negative emotional outcomes other than depression. However, it is likely that mindfulness will help to protect from anxiety as well, given that neuroticism is strongly associated with anxiety disorders as well as mood disorders.

## Figures and Tables

**Table 1:** Summary of intercorrelations, means and standard deviations for scores on EPQ-neuroticism scale, BDI-II and FFMQ.

		1	2	3	4	5	6	7	*M*	*SD*	POMP *M*	POMP *SD*	Cronbach’s α
1.	EPQ-N								14.8	7.2	64.5	31.5	.94
2.	BDI-II	.56⁎⁎							13.5	10.7	24.4	17.1	.93
3.	FFMQ nonreactivity	−.43⁎⁎	−.34⁎⁎						20.1	5.4	57.5	15.4	.81
4.	FFMQ observing	.07	.05	.08					24.3	5.5	60.9	13.7	.76
5.	FFMQ acting w/o awareness	−.51⁎⁎	−.57⁎⁎	.37⁎⁎	−.05				25.8	6.4	64.5	16.1	.90
6.	FFMQ describing	−.29⁎⁎	−.31⁎⁎	.23⁎⁎	.06	.44⁎⁎			25.6	7.0	64.0	17.6	.91
7.	FFMQ nonjudging	−.62⁎⁎	−.56⁎⁎	.41⁎⁎	−.17⁎	.54⁎⁎	.37⁎⁎		24.9	7.6	62.2	19.0	.90
8.	FFMQ sumscore	−.60⁎⁎	−.58⁎⁎	.64⁎⁎	.19⁎	.77⁎⁎	.71⁎⁎	.75⁎⁎	120.9	20.6	62.0	10.6	.90

*Note*. POMP = percent of maximum possible score; EPQ-N = Eysenck personality questionnaire-neuroticism scale; BDI-II = Beck depression inventory II; FFMQ nonreactivity = FFMQ nonreactivity to inner experience subscale; FFMQ observing = FFMQ observing subscale; FFMQ acting w/o awareness = FFMQ acting without awareness subscale; FFMQ describing = FFMQ describing subscale; FFMQ nonjudging = FFMQ nonjudging of experience subscale.

⁎ *p* < .05.

⁎⁎ *p* < .01.
